# Surgeons and preventive health: a mixed methods study of current practice, beliefs and attitudes influencing health promotion activities amongst public hospital surgeons

**DOI:** 10.1186/s12913-019-4186-y

**Published:** 2019-06-06

**Authors:** Stephen Barrett, Stephen Begg, Andrea Sloane, Michael Kingsley

**Affiliations:** 1 Bendigo Health, Bendigo, Victoria 3552 Australia; 2 La Trobe Rural Health School, La Trobe University, PO Box 199, Bendigo, Victoria 3552 Australia

**Keywords:** Surgeons, Health promotion, Professional practice, Hospitals, Attitude

## Abstract

**Background:**

Little is known about the participation of surgeons in preventative health activities in the non-admitted hospital care setting. The aim of this study was to identify which preventive health activities surgeons practice and to explore their attitudes towards preventive health.

**Methods:**

A mixed methods study was conducted using a sequential explanatory design. Quantitative results were obtained from a self-reported clinician survey (*n* = 16) and a Generalized Estimating Equation was used to assess the relationship between dependent (preventive health practice) and independent (confidence and knowledge in preventive health practice, years of practice, and attitudinal factors) variables. Using a building approach to integration, results from the quantitative analyses informed design of the interview guide. Surgeons’ beliefs and attitudes were explored using in-depth, semi structured interviews with a purposeful sample of surgeons (*n* = 14). Responses were collected, independently coded and analysed using a qualitative descriptive approach.

**Results:**

In accordance with a contiguous narrative approach to integration, the quantitative and qualitative findings are reported separately. The clinician survey found that the surgeons carried out preventive health activities at low levels. Preventive health advice was predominantly verbal in nature, and few surgeons provided written material or referred patients to additional services. The GEE analyses indicated that the following factors best predicted the tendency to undertake preventive health activities: years of clinical practice (*p* = 0.041), and the perceived work priority placed on preventive health (*p* = 0.008). Interviews generated four themes that influenced the tendency of surgeons to undertake preventive health activities: perceptions of their role in preventive health, perceived motivation of patients, hospital structure, and facilitating factors. In regards to enabling factors that are likely to increase preventive health practice, surgeons unanimously advocated for referral pathways into specialist behaviour change programs that they could facilitate within their relatively brief consulting time.

**Conclusions:**

The findings suggests that the majority of public hospital surgeons engage in routine preventive health advice at a low level. The high volume of non-admitted surgical consultations undertaken annually, coupled with medium to high self-reported knowledge and confidence in addressing behavioural risk factors, support an increased involvement of surgeons in preventive health practice.

**Electronic supplementary material:**

The online version of this article (10.1186/s12913-019-4186-y) contains supplementary material, which is available to authorized users.

## Background

Chronic non-communicable diseases are the foremost cause of preventable illness, disability and death worldwide [1]. Smoking, diet, and insufficient physical activity are the primary behavioural risk factors behind preventable chronic diseases [2]. The increased prevalence of chronic diseases has influenced demands on the health system [3], with chronic diseases leading to hospitalisations, long-term disability, and rehabilitation costs [4]. Accordingly, hospitals need to broaden their role from their primary focus on disease treatment towards a position of more integrated health promotion [5].

Hospitals are well situated to play a key role in the delivery of preventive health [5–8]. As hospital clinicians, surgeons have an important role in advocating for behaviour change for patients with, or at risk of, chronic disease [9]. Due to their extensive medical training and specialisation, surgeons are regarded as reliable sources of medical advice, extending beyond their expertise in surgical care [6]. Surgery is considered a major life event [10], and individuals are more susceptible to behaviour change in the face of such an event [11]. Surgeons therefore, have potential to be influential in the promotion of lifestyle behaviour change [12]. Surgeons undertake high volumes of non-admitted consultations annually, which provides opportunities to address preventive health directly during routine clinical interactions [13]. In Australian hospitals alone, over 2.2 million elective admissions involving surgery were undertaken in 2015–2016 [14, 15]. In spite of this, there is a scarcity of research investigating preventive health practice in non-admitted surgical practice.

Studies examining lifestyle risk management (smoking cessation and/or physical activity promotion) delivered by hospital doctors have consisted largely of cross-sectional studies of self-reported practice [12, 16–20]. Only two of these studies, which both focused on oncology patients, included hospital surgeons [16, 20]. Findings in all studies demonstrated low rates of preventive health interventions [12, 16–20]. In addition, hospital doctors report low levels of confidence in their ability to assist patients with health behaviour change [17, 19] and uncertainty over the effectiveness of behaviour change advice [16]. In the studies [12, 16–20], no interviews were carried out to probe the survey findings and understand the beliefs and attitudes that might explain the low levels of preventive health interventions undertaken.

Given the prevalence of chronic disease and the necessity for hospitals to move to a position of more integrated preventive health practice [5, 8, 21, 22], it is important to gain insights from hospital surgeons due to the influence they may exert on patient behaviour [12]. Surgeons are clinical leaders with responsibility for clinical performance as well as clinical policy and practice [23]. As such, surgeons maintain autonomy over practice standards [23], and little is known about the opinions of this professional group concerning preventive health practice. The depth of insight gained from the study of surgeons might offer distinctive perspectives on current preventive health practice and the attitudes and beliefs of these highly professionalised clinicians relating to implementing preventive health into non-admitted surgical practice. Therefore, the aim of this study was to identify which preventive health activities surgeons carry out in non-admitted public hospital clinics and to explore the attitudes of the profession towards preventive health practice.

## Methods

**Fig. 1:**
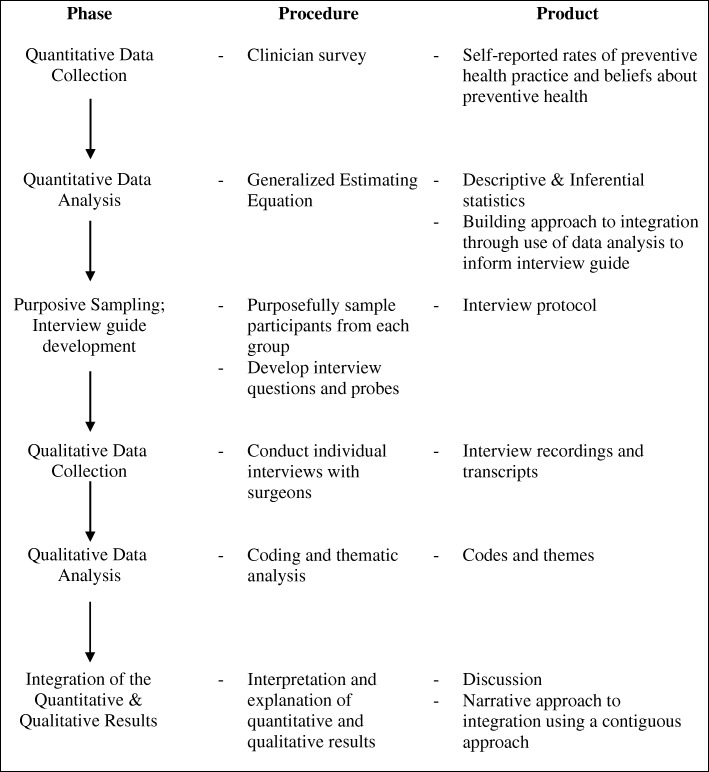
Mixed-methods integration flow diagram

This study used a mixed-methods design to identify which preventive health activities surgeons carry out in non-admitted public hospital clinics and to explore the attitudes of the profession towards preventive health practice. We integrated mixed-methods at the design level using a sequential explanatory design [24–26]. This two-stage design began with a self-reported clinician survey investigating surgeons’ actual participation in preventive health activities (Fig. 1). This was followed by the subsequent collection and analysis of in-depth interviews with surgeons to gain insight into the attitudes of surgeons towards undertaking preventive health activities in non-admitted settings. The protocol for this study has been detailed previously [27]. Ethical approval for the study was gained from the human research and ethics committee of the participating hospital and the associated university.

### Participants

This study targeted all surgeons and their registrars consulting in an elective outpatient clinic of a major tertiary hospital in regional Australia. Participation was offered to all practicing surgeons (general and orthopaedic; *n* = 20) and registrars (*n* = 11) between June 2017 and August 2018. The recruitment strategy has been described elsewhere [27]. In brief, an email containing the link to the clinician survey was sent to all potential participants by their head of department. Participants were informed prior to commencing the survey that informed consent was implied by completing the survey. For the interviews, surgeons were approached individually, in the non-admitted clinic by a project officer to discuss participation. Informed consent was sought from all participants prior to completing the interview.

### Clinician survey

The clinician survey requested detail on surgical practice, including surgical speciality, and number of year of practice. Participants were asked to self-report on proportions of patients who they screened for behavioural risk factors (smoking, diet, physical activity and alcohol), provided verbal and/or written advice and referred to other services for support in changing risk factors (Additional file 1). The survey measured surgeons’ knowledge and confidence in screening and managing risk factors, as well as attitudinal measures relating to the delivery of preventive health interventions in surgical care. All survey items were measured on a 5-point Likert scale. Data were analysed using IBM SPSS Statistics for Windows (Version 25; IBM Corp., USA).

### Semi-structured interviews

Following analysis of the clinician survey, face-to-face interviews were conducted with a purposeful sample of surgeons and registrars (*n* = 14). Maximum variation sampling was used to ensure that a heterogeneous sample was recruited, to capture the perspectives of orthopaedic and general surgeons and registrars to search for variation in perspectives [28]. In total, 21 surgeons were asked to participate in the interviews, with a participation rate of 71%. We employed a building approach to mixed-methods integration, using the results from our quantitative analyses to inform the data collection of the qualitative component [25, 26]. Quantitative data were used to develop the interview guide (Table 1). Interviews were conducted by the first author and covered issues related to preventive health practice in routine practice and the attitudes of surgeons towards preventive health practice. All interviews were audio-taped with participants’ permission and transcribed verbatim by the first author for thematic analysis [29]. Field notes were used to supplement the audio and transcripts to inform the iterative development of interview guides and question-related probes for subsequent interviews.

**Table 1:** Interview guide for surgeon interviews with rationale for questions

Domain	Relevant quantitative findings	Interview question	Rationale for the question
Overview of clinical practice	● NA	1. Using a category 2 or 3 patient (expected wait to surgery between 90 and 365 days) as an example, can you please give an overview of a routine clinical consult?	○ Elicit from the surgeons, in their own words, what constitutes routine practice in the non-admitted setting.
		2. We are particularly interested in the steps between telling the patient they need the procedure and the end of the consultation- do you spend any time discussing what the patient could do in this waiting time?	○ Elicit from the surgeons whether preventive health discussions arise with patients in non-admitted practice.
Exploration of survey results	● How important surgeons felt it was to address lifestyle changes with patients was independently associated with preventive health practice rates (p = 0.006). This factor did not contribute to the model that best predicted preventive health practice (*p* = 0.056).	1. From the clinical survey of practicing surgeons, the vast majority of surgeons indicated that addressing behavioural risk factors is important for health. At the same time however, the rates of implementation amongst the sample was low to medium. Have you any thoughts about this?	○ Elicit opinion from surgeons as to why, despite acknowledging the importance of addressing lifestyle changes with patients, preventive health practice was predominantly undertaken at low levels.
	● Independent associations were observed between with preventive health practice rates and surgeons’ confidence (p = 0.008) and knowledge (*p* = 0.029) at addressing lifestyle changes. Neither confidence (*p* = 0.184 and knowledge (*p* = 0.543) contributed to the model that best predicted preventive health practice.	1. Again from the survey, surgeons indicated medium to high levels of confidence/knowledge in addressing behavioural risk factors; what we found interesting was, despite this perceived confidence/knowledge, a very low number of respondents carried out preventive health interventions. Have you any thoughts about this?	○ Elicit opinions from surgeons as to why, despite reporting medium to high levels of confidence/knowledge in addressing behavioural risk factors, preventive health practice is predominantly undertaken at low levels.
Attitudes to preventive health	● How much of a work priority surgeons place on addressing lifestyle changes with patients significantly predicted tendency to undertake preventive health interventions (β = 1.22, p = 0.008).	1. Do you think it is an appropriate part of your job to be spending time with patients on preventive health?	○ Elicit opinions from surgeons as to the association between work priority and preventive health practice.
	● The GEE model found two factors that together, significantly predicted tendency to undertake preventive health interventions, including number of years of clinical practice (β = 0.26, p = 0.041) and work priority (β = 1.22, *p* = 0.008).	1. What are some reasons for deciding to engage in preventive health practice with your patients?	○ Elicit rationale from surgeons for their engagement in preventive health.
		2. On the other side, what are some reasons for deciding not to engage in preventive health practice with your patients?	○ Elicit rationale from surgeons for their non-engagement in preventive health.
Working environment	● NA	1. Time is a known barrier to undertaking health promotion in routine work, this is well established. The Specialist Clinic is extremely busy, and unlikely to see changes in time demands. At the same time public health institutions continue to call on doctors to do more. In the absence of more time, what can be done to facilitate this?	○ Elicit opinions from surgeons in relation to the call for hospitals to integrated preventive health into routine care.
Future directions	● N/A	1. What might need to be done differently in order to increase delivery of health promotion interventions?	○ Elicit opinions from surgeons as to the potential to change preventive health practice rates in non-admitted settings.

### Analyses

From the clinician survey, surgeons’ implementation rates in preventive health activities (assessing risk factors, proving information and making referrals) were classified as high, medium or low [30]. High implementation rates defined screening and/or intervention scores in the fourth quartile for responding surgeons. Low implementation rates defined screening and/or intervention scores less than or equal to the first quartile for responding surgeons. Quartile cut-off points were also included for surgeon confidence, knowledge, and attitudinal measures.

Spearman’s rank-order correlations were performed to assess the relationships between the dependent variables (preventive health practice) and independent variables (confidence and knowledge in preventive health practice, years of practice, and attitudinal factors). Following this, a Generalized Estimating Equation (GEE) was used to model the associations between independent variables and preventive health practice [31]. The GEE indicates which variables, when added to the model, best predict preventive health practice. Goodness of fit for the GEE model was assessed using the quasi-likelihood under independence model criterion (QIC) [32]. The QIC is a statistic for model selection for GEE models, where lower values indicate better model fit to the data [32].

Data from in-depth interviews were collected and analysed concurrently. Qualitative description was used as the theoretical framework for the qualitative component [29]. Qualitative description provides straightforward, rich descriptions of experiences or events in a language similar to the participant’s own [29]. Transcribed transcripts were analysed and coded line-by-line using the qualitative data analysis software NVivo 10.0 (QSR International, Cambridge, MA, USA). Codes were derived from data rather than being determined beforehand, and a coding scheme was applied to the interview text. Coded text was grouped into more general categories, which were reviewed by the research team and merged into themes to help explain the factors that influence surgeons’ participation in health promotion activities [33, 34]. Two authors (SB^1^ and AS) independently coded and analysed the data. To improve reliability and to reach consensus, two additional authors (MK and SB^2^) reviewed the codebook and samples of transcripts. No new information was found between the twelfth and thirteenth interview, indicating that data saturation was reached by the twelfth interview [37]. To ensure data saturation, one additional participant was interviewed. As this additional interview did not bring forward new information, data saturation was deemed to have occurred [37], and interviewing was ceased.

## Results

In total, 16 surgeons completed the survey (response rate of 51%) and interviews were carried out with 14 surgeons (participation rate 71%). The surgeons that participated in the interviews were broadly representative of those completing the survey (Table 2). The majority of surgeons worked full-time, and three quarters of surgeons in both the survey and interviews were male. The results of the quantitative and qualitative components are reported in separate sections, using a contiguous narrative approach to integration of mixed-methods data [26].

**Table 2:** Characteristics of the surgeons participating in the survey and interview

	Survey (*n* = 16)	Interviews (*n* = 14)
Surgeon Type, No (%)		
General surgeon	5 (31%)	6 (43%)
Orthopaedic surgeon	4 (25%)	4 (29%)
Registrar- general surgery	4 (25%)	3 (21%)
Registrar- orthopaedic surgery	3 (19%)	1 (7%)
Gender, No. (%)		
Female	4 (25%)	5 (36%)
Male	12 (75%)	9 (64%)
Employment, No (%)		
Full time	15 (93%)	14 (100%)

### Clinician survey

Table 3 provides preventive health practice rates and attitudes to preventive health amongst the responding surgeons. Overall, all surgeons carried out some preventive health activities, however the majority of surgeons did this at low levels. Asking patients about behavioural risk factors and providing verbal advice were the most undertaken preventive health interventions. Only 2 surgeons reported providing patients with written advice, and 3 surgeons reported having referred patients to other service providers for help with risk factor management. The surgeons self-reported knowledge and confidence in addressing behavioural risk factors was medium to high.

**Table 3:** Self-reported rates of preventive health practice and attitudes to preventive health amongst survey respondents (*N* = 16)

Variable	High^a^	Medium^a^	Low^a^	No Activity^b^
	Number (proportion)			
Preventive health activities				
Overall preventive health practice rates	1 (6%)	3 (19%)	12 (75%)	0
Asking patients about behavioural risk factors	2 (12%)	4 (25%)	10 (63%)	0
Assess patients readiness to change their behaviour	0	5 (31%)	9 (56%)	2 (12%)
Provide verbal advice to patients	2 (12%)	4 (25%)	10 (63%)	0
Provide written advice to patients	0	0	2 (12%)	14 (88%)
Refer patients to other service for help in managing their risk factor	0	0	3 (19%)	13 (82%)
Attitudes to preventive health				
Confidence in addressing lifestyle changes	6 (38%)	9 (56%)	1 (6%)	–
Knowledge in addressing lifestyle changes	5 (31%)	10 (63%)	1 (6%)	–
How effective you think your advice is in helping clients with lifestyle changes	0	10 (63%)	6 (37%)	–
Patients find it agreeable for me to raise lifestyle changes as part of consultation	0	12 (75%)	4 (25%)	–
How important lifestyle changes are for health	11 (69%)	5 (31%)	0	–
How important it is to address lifestyle changes with patients	7 (44%)	9 (56%)	0	–
How much of a work priority is it to address lifestyle changes with patients	1 (6%)	13 (82%)	2 (12%)	–

^a^ High implementation rates defined screening and/or intervention scores in the fourth quartile for responding surgeons. Low implementation rates defined screening and/or intervention scores less than or equal to the first quartile for responding surgeons. The same quartile cut-off points are used for attitudes to preventive health

^b^ Scores of 0 for rates of preventive health activities

In the Spearman’s correlation, significant positive correlations were observed between preventive health practice and clinician confidence (r = 0.635, *p* = 0.008), knowledge (r = 0.544, *p* = 0.029), perceived effectiveness of preventive health practice (r = 0.710, *p* = 0.002), the importance placed on addressing lifestyle changes (r = 0.655, *p* = 0.006), and the work priority placed on addressing lifestyle changes with patients (r = 0.644, *p* = 0.007). The GEE model found two factors that together, significantly predicted tendency to undertake preventive health interventions, including number of years of clinical practice (β = 0.26, *p* = 0.041) and work priority (β = 1.22, p = 0.008) (Table 4). The addition of work priority to the model decreased the QIC from 1063 to 736, indicating a more robust fit of data to the model. The lower QIC indicates that the model, with the addition of work priority, contains the best subset of explanatory variables to predict the surgeons undertaking of preventive health interventions.

**Table 4:** Statistical analyses for variables predicting tendency to undertake preventive health activities (N = 16)

	Spearman’s		Generalized Estimating Equation	
	*Bivariate correlations*		*Parameter Estimates*	
Variable	*RO*	*p*	*β*	*p*
Confidence in addressing lifestyle changes	0.635	0.008	0.386	0.184
Knowledge in addressing lifestyle changes	0.544	0.029	−0.193	0.543
How effective you think your advice is in helping clients with lifestyle changes	0.710	0.002	0.254	0.747
Clients I see find it agreeable for me to raise lifestyle changes as part of consultation	0.180	0.505	−0.305	0.261
How important lifestyle changes are for health	0.134	0.620	−0.008	0.990
How important it is to address lifestyle changes with patients	0.655	0.006	1.159	0.057
How much of a work priority is it to address lifestyle changes with patients	0.644	0.007	1.217	0.008
How many years of clinical practice have you undertaken?	0.368	0.164	0.258	0.041

Dependent Variable: Implementation of preventive health interventions

GEE Model: (Intercept), Time, Confidence, Knowledge, Effectiveness, Agreeable, Important for health, Important to address, Work priority, Years of clinical practice

Quasi-likelihood under independence model criterion (QIC) = 736

### In-depth interviews

Four themes were found to influence surgeons’ preventive health practice. The themes, which all centred around the clinical consultation, included: surgeon’s perceptions of their role in preventive health, perceived motivation of patients, the hospital structure, and facilitating factors. The codes, categories and themes are described in Additional file 2. These themes are expanded upon below using verbatim quotes from participants for illustrative purposes. Additional verbatim quotes for each theme are provided in Additional file 3.

#### The role of the surgeon in preventive health

All surgeons considered preventive health to be important for health. However, the perceived importance did not translate to high rates of preventive health practice. Surgeons who reported undertaking behaviour change discussions with patients reported that their role was to address behaviour change in relation to specific surgical practice, rather than a holistic wellbeing perspective; for example, smoking cessation was advocated to decrease the risk of infection.

*“I’ll be telling them to either cut back on the smoking or try to aim quitting if it’s possible, at least for the surgery, and then after that if they can continue then great; if they can’t then at least for the time period for the surgery if they can do that that would be great” (Surgeon 3).*

Some surgeons felt that behaviour change is not part of the role of the surgeon; that surgeons are clinical specialists who have a priority to treat specialist problems, thereby delivering services that no other clinician can, as suggested by the following quote:

*“So we know what you need to do, but you know, we are trained to do surgery. And other people can’t do that, and that’s what we need to do. If you make us do all of this other stuff, then it’s not a particularly effective use of surgeon time” (Surgeon 9).*

Viewing themselves as specialist practitioners, surgeons believe their role is best suited to focusing on presenting conditions, rather than taking a holistic approach to the person.

*“Probably because surgeons don’t feel that it is their job to do that; they are referred a patient for a [specific problem], and they are concentrating on treating that … . they probably zoom in on that pathology rather than looking at the patient as a whole” (Surgeon 13).*

### The motivation of the patients

Patient motivation and acceptance of behaviour change interventions by the patient was another important theme. The surgeons described patient acceptance of lifestyle interventions as an important factor that influenced the surgeons’ participation in preventive health practice. Consistent with existing literature [35], some surgeons reported that patients are not opposed to surgeons raising lifestyle discussions during consultations. This advice however, doesn’t necessarily translate to actual behaviour change, with surgeons reporting that many patients maintain their behaviours despite the provision of advice.

*“I think a good percentage of patients, you say ‘you need to quit smoking or if you don’t, your risk of infection is higher’ and they are like, ‘ah yeah, whatever’ and you see plenty where you have no response” (Surgeon 1).*

Surgeons highlighted that the persistence of risky health behaviour by patients despite health advice was a source of frustration, which might decrease the likelihood of surgeons engaging in preventive health activities in the non-admitted setting.

*“And we say ‘you have to cut down smoking’ and every time they come back to clinic and we go ‘have you cut down on smoking?’ and they are still smoking. I think after a while, you just … you are talking to a brick wall” (Surgeon 3).*

*“I mean smoking we all know in particular when it comes to wound healing and infection, that’s something we all know is not good. However, I don’t always advise them to stop because, I don’t know, I often don’t think they will stop” (Surgeon 6)*

The surgeons reported that not all patients are appreciative of discussing their lifestyles during surgical consultations. In the surgeons’ experience, many patients attend the consultation seeking specialist advice relating to a particular issue, and are not seeking generalist advice about health behaviours. Previous experience of negative patient reactions may contribute to the surgeons’ narrowing the focus of the consultation to that of the presenting condition only.

*“ … most people don’t want to talk to a surgeon in an outpatient clinic about their overall wellbeing. They have come here for a problem, so it [consultation] needs to be problem-focused” (Surgeon 12).*

### The hospital structure

Surgeons work in busy public hospital clinics that have extensive waiting lists. Surgeons are responsible for clinic throughput, and are accountable to management on such performance indicators. The pressure for volume is a barrier to holistic care. Working under time pressure, curative care is prioritised over preventive health. This issue is compounded by the complexity of the hospital system where there is a disconnect between the absence of preventive health in outpatient clinics and sub-optimal post-operative surgical outcomes.

*“ … the holistic approach, then probably my extra 5 minutes doing that referral is in the patient’s best interest, I get that. But hospitals don’t always look at the whole picture, they look at the bottom line for them; so the people in clinic here, running the clinic, will be looking at their targets … they don’t really care what they spend up on the surgical ward when the patient gets a wound infection that might be preventable if they weren’t a smoker” (Surgeon 13).*

One surgeon changed their practice by limiting the number of patients seen in the clinic, allowing increased time with patients.

*“We have limited it to 18 patients between the 2 of us, and so it’s good, there is enough time if you have enough reviews which are quick, and ‘new’ which are not. Yes, there is enough time [for preventive health]” (Surgeon 7).*

This surgeon acknowledged that, as a senior hospital clinician, the clinical and institutional influence afforded to him may have permitted such a policy change, but this is ultimately at the discretion of hospital administrators.

With respect to generating referrals, surgeons highlighted an absence of specific programs for general behaviour change in the hospital, as well as poor awareness of behaviour change programs in the community.

*“For people who have been inpatients there [are] options, but there aren’t a lot of things for just young inactive people unfortunately” (Surgeon 12).*

*“I don’t know how you would actually make the referral [to community programs]” (Surgeon 2).*

Surgeons who refer to internal services, such as exercise physiologists and physiotherapy, use this pathway to address issues that relate to surgical outcomes, increasing muscle strength before surgery for example, rather than increasing physical activity for general health. Surgeons also forego preventive referrals to allied health practitioners due to the demand for rehabilitation services.

*“ … from a public health system, it’s hard to get them involved in exercise programs. Physiotherapists are often very busy and overworked, and they can’t just be doing exercises with them” (Surgeon 11).*

### Facilitators experienced by surgeons

Surgeons were unanimous in their desire for information to give to patients that are specific to their needs (i.e., smoking specific or physical activity specific). The majority of surgeons felt that a referral pathway into specialist behaviour change services, either internally or externally is required to facilitate successful behaviour change.

*“A flyer would be good … . and I say put that on your fridge, something like that, where you see it every day, and you think ‘oh, the specialist gave it to me’” (Surgeon 8).*

The surgeons felt that if they had dedicated resources, or referral pathways to offer patients, then they could use their clinical influence to stress the importance of behaviour change, which might increase the likelihood of patients using these services.

*“I think a clinician handing it [referral] to them, and underlying who they need to see would be much more effective” (Surgeon 13).*

*“We need to be able to say ‘you need to make this change, here is someone who can help’. But it comes from the surgeon as the authorizing environment” (Surgeon 12).*

## Discussion

This mixed-methods study identified which preventive health activities surgeons carry out in non-admitted public hospital clinics, and explored the attitudes of these professionals towards preventive health practice. The quantitative data suggests that surgeons carried out preventive health interventions at low levels. Face-to-face conversations with patients about behavioural risk factors was the most commonly undertaken intervention. Surgeons were unlikely to provide written advice or refer patients to additional health behaviour change services. Although a number of attitudinal factors individually correlated with rates of preventive health practice undertaken, collectively, years of practice and the work priority placed on addressing lifestyle change were the strongest predictors of preventive health practice identified in the quantitative analyses. The qualitative analysis identified several individual and institutional topics that influenced surgeons undertaking of preventive health practice in non-admitted clinical care, with surgeons preferencing referral pathways into specialist programs to assist patients with behaviour change.

In contrast to previous research [16, 17, 19], lack of knowledge or confidence were not identified as barriers to preventive health practice. Although surgeons’ knowledge and confidence were independently associated with levels of preventive health practice in the quantitative analyses, these variables did not contribute to the model that best predicted preventive health practice rates. The quantitative analysis also highlighted that how important surgeons believe it is to address lifestyle changes with patients was independently associated with preventive health practice in the quantitative analyses, though this did not contribute to the model that best predicted preventive health practice rates either. Quantitative data suggests that although surgeons believe it is important to address lifestyle changes with patients, and are confident and knowledgeable in doing so, these factors do not predict actual rates of preventive health practice. This might reflect the medically oriented work of surgeons, and that surgeons, rather than lacking confidence or knowledge, do not see preventive health as core to their role [36], which was probed in the subsequent qualitative interviews. The surgeons endorsed this biomedical perspective in the qualitative interviews, preferring to practice under a scope of vision restricted to the presenting issue. Surgeons’ engagement in health discussions predominantly relate to surgical outcomes; they did not consider it part of the surgical role to discuss general wellbeing.

In the qualitative interviews, discussions with patients about smoking cessation was the most commonly noted preventive health topic; however, as exemplified by the quotes, smoking cessation was advised due to the operative risk, not for general health. Surgeons’ engagement in risk mitigation through smoking cessation advice is likely to be influenced by the well-publicised literature relating to smoking and post-operative risks [37, 38]. Despite unequivocal evidence that behaviour change interventions are effective in multiple settings [39, 40], few interventions have targeted hospital surgical patients [41] and very few studies have addressed behaviour change in non-admitted surgical clinics [42]. The lack of published literature on surgeons’ preventive health practice might influence the surgeons’ perception that preventive health does not fit within their role.

In the quantitative analyses, surgeons’ perceptions regarding patient acceptance of lifestyle interventions in non-admitted care was not significantly correlated with preventive health practice rates. The acceptance of lifestyle interventions on the part of the patients, and the patients’ motivation to undertake behaviour change was however, repeatedly brought up in the qualitative interviews. Many surgeons reported that although patients are agreeable to receiving lifestyle advice during a non-admitted surgical consult, the provision of advice did not translate to actual behaviour change on the part of the patient. On the other hand, surgeons noted that many patients attend specialist appointments seeking specialist advice, and are not interested in, or motivated by the provision of preventive health in non-admitted surgical care. From the interviews, differences were observed between surgeons as to whether they attempted to use the consultation to illicit patients’ motivation to change. Discussion of risk factors is standard practice for surgeons [43] and in the interviews some surgeons reported using the consultation as an opportunity to link behavioural risk factors to the presenting health issue. Opportunistic health promotion is strongly advocated in chronic disease prevention and management [44]. The quantitative analyses indicated that surgeons who prioritised preventive health were significantly more likely to use the clinical opportunity to undertake preventive health interventions.

Not all surgeons however, approached the consultation as a chance to motivate patients, with some surgeons expressing concern about engaging in preventive health discussions with patients in the non-admitted setting. These concerns might reflect a didactic understanding of preventive health, where the passive patient is expected to adhere to the prescriptions of the healthcare expert [45]. Alternative models exist, however, where emphasis is placed on empowering patients over their own health, rather than delivering purely instructive messages [45]. The interviewed surgeons, ambivalent about engaging in behaviour change discussions should be encouraged by research indicating that the majority of patients view hospitals as an appropriate setting for health promotion [35].

Consistent with existing literature, insufficient time was identified as a major barrier to preventive health [16, 17]. Time pressure is institutionally driven, with surgeons under pressure for clinical performance. Surgeons were cognisant of the waiting lists for public services, and the pressure that this places on clinical throughput. Performing under a fixed amount of time, surgeons largely felt that they could not afford to forego time spent in their expert role. In the quantitative analyses, work priority afforded to preventive health was the strongest predictive factor for engaging in preventive health practice, and this finding was subsequently probed in the qualitative interviews. It is important that the surgical profession recognises the role that surgeons can play in preventive health, even in the face of time demands. As little as 3 min of advice can markedly increase a patient’s chance of smoking cessation [46]. The number of years of practice was also a significant predictive factor for engaging in preventive health practice. The qualitative interviews highlighted that one senior surgeon chose to decrease clinical volume, even in the face of service demand. This attests to the aforementioned influence that surgeons might have over institutional practice, and strengthens the argument to engage with surgeons on health promotion policies in the future.

The facilitative topics raised by surgeons in the qualitative interviews were unanimous, with surgeons preferencing pathways to refer patients into specific programs tailored for health behaviour change as a means to facilitate preventive health interventions in non-admitted clinical care. The interviewed surgeons believe that information fliers and standardised referral pathways would allow them to engage, in a time-efficient manner, in preventive health with patients, and subsequently offer follow-on services. The provision of dedicated information, as well as referral pathways could offer avenues for surgeons to integrate preventive health into non-admitted care. Further to this, the development of linkages to community-based aftercare resources is likely to improve continuity of patient care [17], particularly when initiation of behaviour change is driven from the surgical consult [17].

### Limitations

This study is subject to a number of limitations. First, preventive practices were assessed via self-report. This approach is consistent with numerous previous studies, however, the accuracy in assessing actual behaviour is unclear. Second, although our response rate of 51% was higher than other preventive health research with hospital doctors [16, 17], the response rate is lower than observed in studies of surgeons’ clinical decision making [47]. While low response rates can increase the possibility of response bias, significant differences were not observed between responding and non-responding doctors in cross-sectional studies [48]. The doctors studied by Kellermen et al., were physicians, and not surgeons, which might limit the generalizability of the findings [48]. Third, the GEE model might be underpowered to show statistical significance in the majority of measured variables [20]. Further research with a larger cohort of participants might result in differing models that best predict preventive health practice by surgeons [20]. Fourth, a degree of selection bias could have resulted from the survey non-response rates and interview non-participation rates, with the participating surgeons potentially more engaged in preventive health than non-responders [49]. However, surgeons were purposefully sampled for the interviews to ensure a variation in the types of specialty areas and experience to providing insights from multiple perspectives. Finally, this study was undertaken in a single hospital. While single-site studies might limit generalizability, the primary aim of this research was to acquire detailed knowledge about context and processes of the studied phenomenon. Steps were taken to maximize rigour and attain theoretical saturation [27] and these should ensure the broad applicability of the findings to other non-admitted, public hospital services.

### Implications for clinical practice and future research

Surgeons undertaking of preventive health activities is influenced by a multitude of factors, with the working structure of the hospital most likely to influence preventive health practice rates [6, 8]. Heavy workload emerged as a core barrier that cannot be ignored ([36, 50]). The interviewed surgeons were cognisant of the demands of the clinic, and report practising under a narrow specialist approach, foregoing holistic care. Management support is critical for the availability of time and resources required for surgeons to broaden their practice to increase preventive health practice rates [36].

The interviewed surgeons suggested that in order to increase engagement in preventive health activities in non-admitted care, while managing consultation time, their preference was for the creation of information fliers on behaviour change to give to patients, and for referral pathways that link patients to specialist behaviour change programs available either in-house or in the community.

The sheer volume of non-admitted surgical consultations provided annually offers vast potential for opportunistic preventive health in the non-admitted clinical setting [14]. Due to the influence surgeons can exert over patients, it would be valuable to examine how surgeon-initiated referrals to tailored behaviour change programs could be implemented into routine practice, as well as health-related outcomes derived from this pathway.

## Conclusion

This mixed-methods study revealed that the majority of surgeons discuss lifestyle risk factors with their patients at low levels. Surgeons were unlikely to provide written advice or refer patients to ancillary preventive health services. The surgeons largely expressed positive attitudes towards preventive health, and the surgeons who placed the greatest work priority on preventive health were most likely to undertake preventive health practice. To increase preventive health practice, surgeons indicated a preference for pathways to enable referrals into dedicated behaviour change programs that could fit within the scope of non-admitted surgical consultations. Due to the high volume of ambulatory surgical consultations annually, it is important that surgeons remain active participants in preventive health policy.

## Additional files

Additional file 1: Surgeons and Preventative Health Survey. (PDF 542 kb)

Additional file 2: Codes, categories and themes relating to surgeons’ attitudes and beliefs towards the management of lifestyle risk factors. (DOCX 19 kb)

Additional file 3: Themes and corresponding quotes relating to surgeons’ attitudes and beliefs towards the management of lifestyle risk factors. (DOCX 16 kb)

## Data Availability

The datasets used and/or analysed during the current study are available from the corresponding author on reasonable request and with the permission of the relevant Research Ethics Committees.

## Abbreviations

GEE: Generalized Estimating Equation
QIC: Quasi-likelihood under independence model criterion
