# Supplementary material for: Surgeons and preventive health: a mixed methods study of current practice, beliefs and attitudes influencing health promotion activities amongst public hospital surgeons
Source: BMC Health Serv Res. 2019 Jun 6;19:358. doi: 10.1186/s12913-019-4186-y (PMC6555744; doi:10.1186/s12913-019-4186-y)
Supplement: Supplementary file 2 — Codes, categories and themes relating to surgeons’ attitudes and beliefs towards the management of lifestyle risk factors. (DOCX 19 kb) [file 12913_2019_4186_MOESM2_ESM.docx]

**Additional file 2:** Themes and corresponding quotes relating to surgeons’ attitudes and beliefs towards the management of lifestyle risk factors

| **Codes** | **Categories** | **Themes** |
| --- | --- | --- |
| - Time required to do preventive health. - Time available for surgical consultations. - Not trained in this speciality. - The best use of surgeon time. - Opportunity cost. - It is important, but is it my job? - Not why people become surgeons. - Address risk when it has direct influence on surgery/outcomes. - Need to deliver in black and white. - Self-fulfilment. - Role of primary care to manage behaviour. - No follow-up visit to question/reinforce change. - All clinicians have responsibility. | - Is it my role? - Does it fit within my role? - Clinical barriers to preventive health. - Should I do this? - Capacity- how can I do this? | The role of the surgeon in preventive health |
| - Difficult to change behaviours. - Entrained behaviours. - Lots of public health messages out there. - Mass media. - People already know. - Personal choice. - Patient ownership of their issues. - What motivates patients? - Patients want specialist advice- not general advice. - Some things (e.g. smoking) are their only enjoyment. - Maintain behaviours despite advice. - They all claim to do the right things. - Frustrating. - Those who want to change will ask for help. | - Patient behaviours. - Patient understanding (of risk and reward). - Patient beliefs. - Patient preferences. - Will they change? | The motivation of the patients |
| - Time required to do preventive health. - Time pressure per patient in clinic. - Clinic pressures- management. - Answer to management for clinic throughput. - Is it effective- and is it worth my time? - Disconnect between preventive health and surgical outcomes. - Limited/no hospital referral pathways. - Pressure for hospital resources. - Don’t know what’s available in community. - No information to give to patients. | - Institutional practice/ way of working. - Institutional pressures. - Cost effectiveness of adding preventive health. - Trade-off between clinic throughput and optimal practice. - Hospital resource and capacity. - External resources. | The hospital structure |
| - Patients like something tangible. - Give information to patients. - Easy to read handouts. - Information needs to be specific to problem. - Surgeon can sell importance of change. - Links to a specialist clinic/program. - Referrals into specialist change programs. - Short timeframe required. - Efficient use of time. | - Educational material. - Internal referral pathways/capacity. - External referral pathways/capacity. - Time efficient pathways. | Facilitators experienced by surgeons |
