# Supplementary material for: Surgeons and preventive health: a mixed methods study of current practice, beliefs and attitudes influencing health promotion activities amongst public hospital surgeons
Source: BMC Health Serv Res. 2019 Jun 6;19:358. doi: 10.1186/s12913-019-4186-y (PMC6555744; doi:10.1186/s12913-019-4186-y)
Supplement: Supplementary file 3 — Themes and corresponding quotes relating to surgeons’ attitudes and beliefs towards the management of lifestyle risk factors. (DOCX 16 kb) [file 12913_2019_4186_MOESM3_ESM.docx]

Additional file 3: Themes and corresponding quotes relating to surgeons’ attitudes and beliefs towards the management of lifestyle risk factors

| **Theme** | **Quote** |
| --- | --- |
| **The role of the surgeon in preventive health** | *“These patients are being referred to the clinic, despite these problems, so these problems aren’t the reason they are being referred to clinic. If these [SNAP] were the problems they were being referred to clinic for we would be addressing those problems” (Surgeon 3).*  *“So it probably isn’t the best use of our time, but I think it’s still important” (Surgeon 9).*  *“So in a Specialist Clinic where you are seeing them for a specialist problem you try and deal with that [problem] first and foremost, and you hope that the person referring them into the specialist has taken the time to initiate management for these preventative measures, because that’s what we [surgeons] anticipate our GP to be doing for us” (Surgeon 4).*  *“It’s hard to know, I mean, do we have a responsibility to promote that more for their long term management? Yes, probably, but, also we are seeing them from a specialty point of view as well, so it’s hard” (Surgeon 2).*  *“And unless you have a really strong motivator its unlikely to happen. So I think, and if that’s what your bent is [behaviour change], then you won’t have chosen surgery. People who choose surgery choose surgery because they have got a problem, we fix it, and they get better. And that’s not how behaviour change works” (Surgeon 10).*  *“It’s always more difficult to do non-operative management than operative management. Operative is: you have a worn out knee we need to do a knee replacement- these are the risks. Non-operative is you have to change your life” (Surgeon 11).*  *“There’s probably more to gain for us as surgeons as well, if we can get someone to stop smoking or lose weight, you know, who has like complex hernia, it will have a big impact on immediate complications and things for us” (Surgeon 12).*  *“So I have probably 20 patients to see in clinic and I want to get them in and out as quickly as I can because the more I see the better that is; I’m under pressure to see as many patients as I can, and get the turnover and we earn more money for the hospital” (Surgeon 12).*  *“I just think people should not ignore the fact that you know… society is to blame, all this sort of concept that people have, “I should never have been this sick, oh I don’t know why I have cancer”. There is a lot that can be done by them [patients] to improve their own circumstances. And if they don’t have someone throw it up to them particularly someone they don’t know very well… if you have had a long term effort with someone you have known for twenty years, there’s very little impact there” (Surgeon 4).*  *“I think it’s hard, it’s hard for any doctor to know what to advise people on because there isn’t a one size fits all. And we don’t get, we don’t get the education ourselves, because there is so much confusion in the literature, so you can’t, you know, you are going to be constantly chopping and changing, and you know there is so many things you need to know about, you can’t keep up with everything you need to know about [surgery], as well as what vogue lifestyle changing diet are we doing this week” (Surgeon 5).*  *“I mean, I think if we can have an impact, and we know they may listen to us more I think we probably should make more of an effort and address that [preventive health] with every patient we get if they are concerned” (Surgeon 6).*  *“Well I don’t get paid for dealing with all of their other health problems” (Surgeon 12).* |
| **The motivation of the patients** | *“What I have found is a lot of the motivated ones just stop, they just do it on their own. They don’t need us [surgeons], they don’t need anybody else telling them” (Surgeon 3).*  *“So if I see somebody with chronic diarrohea, and I said to them ‘if you stop smoking then your diarrohea will get better’ then I think they would listen to that. But if I’ve got somebody with chronic diarrohea and I give them advice about their chronic diarrohea and I say to them ‘oh yeah, by the way for the benefit of your health in general you should stop smoking’ then I think they will probably go ‘oh yeah, heard it all before’” (Surgeon 13).*  *“Well if I tell people they need to stop smoking for their health, but, for example I will still take out their gall bladder whether they smoke or don’t smoke, they are going to keep smoking. Because there is a reason why they are still smoking, and it’s because they have decided that they like smoking, or they ‘can’t’ stop” (Surgeon 5).*  *“Absolutely. Absolutely. I think they already know, but I think a lot of the time they, sort of, they just continue on anyway. And it’s sort of… it does get emotionally exhausting telling these people over and over again not to do this and not to do that. And it’s almost like it’s ah, the work that you are trying to do to help them is actively coming against that” (Surgeon 7).*  *“I think sometimes, I feel like these days you have to be in modern times, you need to be more careful in the way you word thing to patients; certainly as a junior doctor you know, there is this assumption that you can’t just tell patients… If you have someone come in that is obese and they have got problems with their galls stones and they have high blood pressure and diabetes, the facts are they are obese, they’ve got diabetes- it’s poorly controlled, and they are a smoker. It’s all self-inflicted. But if you read a patient the riot act and said ‘you are overweight, you are eating too much, you are eating the wrong things, you are smoking, you are not looking after yourself and this is why it is happening [to you]. It’s all from things that are self-inflicted’. You would probably get a complaint. And then it would go to an administrator. And then it would come to my boss. And then I would get in strife for it. And I think in modern day times with all the… you can classify it, in the general media everyone is a little more politically correct these days, which is great in a lot of way, but when you are in a busy situation, that’s what patients really need to be told. But if you tell someone, in those hard terms, it will reflect back. And hospitals have a lot of pressure now to be, you know, really patient centred. We have patient centred surveys with how they are happy with their care” (Surgeon 8).*  *“I think, patients, I don’t think at this point in time, patients are, and this is my personal opinion, I don’t think they [patients] are as likely to listen to doctors as they are to listen to the media. I think it’s changed in the last 20 or 30 years” (Surgeon 6).* |
| **The hospital structure** | *“If I am not sort of too busy out there [clinic waiting room] I might be open to discussing it further. Where if you know you have all these sort of things going on in the background, I just don’t think about that [preventative health] part to discuss, because in my mind I’m in this mode of trying to be efficient, so it’s probably that I don’t think about it because of that sort of feeling of wanting to keep things moving” (Surgeon 2).*  *“Apart from physio referrals, and that is mainly for strength based exercises, I don’t really make any referrals to anyone else, to any other external ancillary service. No. if it was an easy process, or there was some sort of pathway but in this service, I wouldn’t even [know how]. Say if I needed to make a referral for hydrotherapy, I wouldn’t know how to do that” (Surgeon 1).*  *“Part of it is knowing what you have at your disposal. And equally knowing, say from a public health system, it’s hard to get them [patients] involved in exercise programs” (Surgeon 11).*  *“Unless there is somebody who has ischemic heart disease, and there are specific programs for rehab; for the average person who just needs more physical activity I don’t know that there is a whole number of funded programs except for like local council initiatives, and then that’s not really my sphere of influence” (Surgeon 12).* |
| **Facilitators experienced by surgeons** | *“A flyer would be good. Because I often like… when I see patients in the 6 months or 12 months I always sort of give them their x-ray slip, and I say put that on your fridge, something like that, where you see it every day, and you think ‘of the specialist gave it to me’ (Surgeon 6).*  *“If it [information] comes from us, and you then emphasise it, you emphasise but it comes from us, the sales is there” (Surgeon 6).*  *“… if we had more clinics to send people to [for behaviour change]” (Surgeon 5).*  *“So maybe there is a role for another clinic, or program such as yourself, or you have a specific program… We [surgeons] probably are time constrained to come extent. To do this properly you need probably 15-20 minutes to do it, and that’s probably longer than we spend with most of our patients. So it probably isn’t the best use of our time, but I think it’s still important.*  *Particularly, as you say, for those ones who would be waiting 6 months or so, then there is time to actually improve on all these things, and they would maybe benefit” (Surgeon 9).*  *“You want to make the most of the expertise that you have, that’s special to you as opposed to [spending time on preventive health]. So if you are trying to address it better in clinic, then I think you need to have people there, for whom that’s their role [preventive health/behaviour change]. Who can sit and go, while you are here, you should go and talk to this person and then they can establish a relationship. If you had a counselor or something” (Surgeon 10).*  *“If there was a defined pathway with information for the patients that says ‘this is your condition and this is the management’. And then tracking. Because part of the management is tracking. And ensuring that patients are engaged” (Surgeon 11).*  *“I wish we had handouts and things that we could give to patients. I don’t have patient information brochures for operations that I refer them for. I don’t have Quit pamphlets.*  *So I don’t know that it’s always the information that’s on it that’s that helpful, but it’s the fact that the physically have something that they have taken away, they have something, they can show it to someone. Prompt more discussions” (Surgeon 12).*  *“No. No I think what would be best is if I did fill out the paperwork for them to go to whatever is available, or that I would know what is available. But I don’t know what is available. I don’t know how to refer them. If I did, and could refer them” (Surgeon 13).* |
